# Germline Mutations and Ancestry in Prostate Cancer

**DOI:** 10.1007/s11912-024-01493-x

**Published:** 2024-01-24

**Authors:** Eudoxie Bataba, Kevin Babcock, Kathryn A. Isensee, Binil Eldhose, Indu Kohaar, Gregory T. Chesnut, Albert Dobi

**Affiliations:** 1 Walter Reed National Military Medical Center, Bethesda, MD 20889 USA; 2 School of Medicine, Uniformed Services University of the Health Sciences, Bethesda, MD 20814 USA; 3 Center for Prostate Disease Research, Murtha Cancer Center Research Program, Department of Surgery at the Uniformed Services University of the Health Sciences, 6720A Rockledge Drive Suite 300, Bethesda, MD 20817 USA; 4 Henry Jackson Foundation for the Advancement of Military Medicine Inc., Bethesda, MD 20817 USA; 5 Cancer Biomarkers Research Group, Division of Cancer Prevention, National Cancer Institute, National Institutes of Health, Rockville, MD 20850 USA

**Keywords:** DNA damage repair, Prostate cancer, Germline mutation, Ancestry

## Abstract

**Purpose of Review:**

Prostate cancer is the most frequently diagnosed non-cutaneous malignancy of men in the USA; notably, the incidence is higher among men of African, followed by European and Asian ancestry. Germline mutations and, in particular, mutations in DNA damage repair genes (DDRGs) have been implicated in the pathogenesis of prostate cancer. This review intends to discuss the implication of ancestry on prostate cancer, specifically in regard to lack of diversity in genomic and genetic databases and the ability of providers to properly counsel patients on the significance of cancer genetic results.

**Recent Findings:**

Ancestral differences in prostate cancer-associated DDRG germline mutations are increasingly recognized. Guidelines for treatment by the National Comprehensive Cancer Network® (NCCN®) support germline testing in certain patients, and a myriad of genetic testing panels for DDRG mutations are now available in clinical practice. However, the consensus among providers on what genes and mutations to include in the genetic tests has evolved from experience from men of European ancestry (EA). Gaps in ancestry-informed clinical practice exist in genetic risk assessment, implementation of screening, counseling, guiding recommendations, treatment, and clinical trial enrollment.

**Summary:**

The lack of diversity in tumor genomic and genetic databases may hinder ancestry-specific disease-predisposing alterations from being discovered and targeted in prostate cancer and, therefore, impede the ability of providers to accurately counsel patients on the significance of cancer genetic test results.

## Introduction and Current Prostate Cancer and Germline Testing Guidelines

Prostate cancer is a major public health problem worldwide. Worldwide, prostate cancer accounts for 29% of new cancer diagnoses and 11% of cancer-related deaths [1]. In the USA, prostate cancer is a prevalent malignancy of men with an estimated 288,300 new cases and with 34,700 predicted deaths in 2023 [2]. Decades of research findings indicate that prostate cancer has a strong genetic component with implications to ancestry. The contribution of inherited ancestral components to the higher prostate cancer incidence and disease aggressiveness among men of African ancestry (AA) compared to men of EA has been evident even in equal-access healthcare settings [3••]. Hereditary germline mutations that impair DNA repair pathways have been implicated in the onset and progression of prostate cancer. These mutations correlate to early onset, aggressive or metastatic disease, and disease severity and could potentially affect treatment response and clinical trial enrollment [4–6]. PARP enzymes are a family of enzymes essential in DNA repair mechanisms. In 2020, the PARP inhibitors olaparib and rucaparib were granted FDA approval for use in metastatic castration-resistant prostate cancers of patients with gene mutations in their homologous recombination repair system [7–9]. Along with these advances, germline testing has emerged as an important part of delivering precision treatment in prostate cancer [10].

The National Comprehensive Cancer Network (NCCN) guidelines for prostate cancer now recommend germline testing for patients with a personal history of prostate cancer in the following scenarios: “1) by personal history of metastatic, regional, very high risk localized or high risk localized prostate cancer. 2) by family history and/or ancestry. Germline testing may also be considered in patients with a personal history of prostate cancer in the following scenarios: 1) by intermediate risk prostate cancer with intraductal/cribriform histology, 2) by prostate cancer and a prior personal history of cancer” [11, 12]. The NCCN recommends testing for the following genes: *ATM*, *BRCA1*, *BRCA2*, *CHEK2*, *HOXB13*, *MLH1*, *MSH2*, *MSH6*, *PALB2*, and *PMS2* (Table 1).

**Table 1:** Genes recommended by NCCN for germline genetic testing

Gene	Gene ID	Chromosome band	Mechanism
BRCA1	672	17q21.31	DNA damage repair
BRCA2	675	13q12.3	DNA damage repair
ATM	472	11q22.3	DNA damage response
PALB2	79728	16p12.2	DNA damage repair
CHEK2	11200	22q12.1	DNA strand break response
HOXB13	10481	17q21-22	Tumor suppressor
MLH1	4292	3p22.2	DNA mismatch repair
MLH2	4436	2p21-p16.3	DNA mismatch repair
MLH6	2956	2p16.3	DNA mismatch repair
PMS2	5395	7p22.1	DNA mismatch repair

However, a debated issue of disease management is the lack of general consensus for testing at an early stage or for men with low-risk and localized disease [13]. These patients often choose to go on active surveillance; unless they have family members that were previously identified as BRCA mutation carriers, then they are considered for germline (BRCA) genetic testing and primary treatment [14].

In response to the need, numerous commercial labs have developed genetic test panels that analyze DDRG mutations and evaluate for hereditary prostate cancer risk. Available assays differ in genes and their evaluated mutations, though the majority of tests include genes from the ten recommended by the NCCN. Indeed, there are variabilities between which genes are included in commercial genetic test panels and some tests do not include HOXB13 from the recommended set [10]. Of note, a comprehensive review of whole genome and exome sequencing between 2010 and 2018 highlighted that only 37% of studies recorded race information, and of those, 14% of genomes sequenced were from black patients, indicating that this population is not adequately represented [15].

## Challenges in Genetic Counseling and Germline Genetic Testing in Clinical Setting

**Fig. 1:**
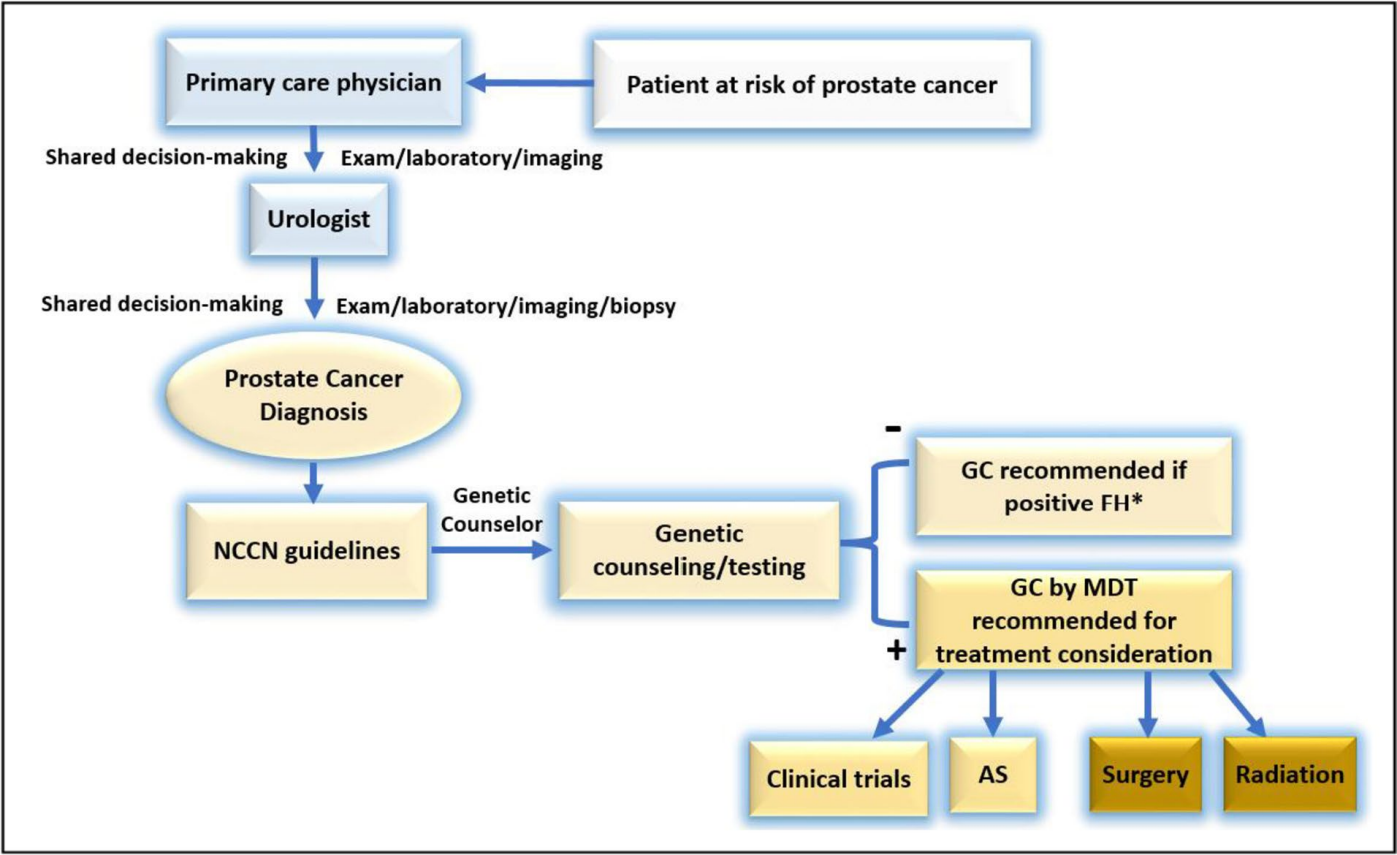
Proposed framework for genetic testing implication in clinical setting (NCCN, National Comprehensive Cancer Network; GC, genetic counseling; FH, family history; MDT, multidisciplinary team; AS, active surveillance; *positive FH per NCCN guidelines)

Genetic counseling is an important aspect of patient care because it enables patients to better understand their disease and how genetics could also impact their families. Given the increased demand for genetic testing for prostate cancer patients, there is also an increased need for providers who understand the role of genetics in the disease process [10]. Giri et al. stressed the critical need to engage and educate primary care providers (PCP) regarding the genetic testing guidelines in prostate cancer [16, 17]. Numerous alternative genetic counseling delivery methods have been suggested including telehealth, video, handout, or pretest counseling done by PCP or urologists. Indeed, genetic counseling is a key step between diagnosis and treatment considerations (Fig. 1).

Suri et al. identified barriers to genetic testing at a single center [18•]. A ten-item survey was administered to a multidisciplinary team consisting of three medical oncologist, three urologic oncologists, two radiation oncologists, a pathologist, and a nurse practitioner. Seventy percent of providers identified the lack of genetic counselors as a barrier, 60% cited lack of knowledge of genetic testing and genetics, and 50% noted confusion about the logistics of the process. Lillard et al. reported that men of EA are more likely to receive genetic profiling earlier in their treatment course compared with patients of AA [15]. These reports highlight the increased need for guidelines and familiarization with the genetic testing process for all providers who evaluate men at risk for prostate cancer, as well as recognition of how ancestry can influence treatment course. Overall, these suggestions aim to improve access to genetic counseling, testing, quality, and equity of care.

The implementation of rapid advances in the personalized management of prostate cancer comes with challenges. Shore et al. emphasized the value of multidisciplinary teams to overcome these difficulties [19]. These teams will be essential in navigating the controversies related to the diagnosis, treatment, and monitoring of prostate cancer. Typical members would include urologists, medical and radiation oncologists, nurses, radiologists/nuclear medicine physicians, pathologists, and genetic counselors, although the composition of the team can vary depending on the center or the severity of disease.

## Ancestral Differences and Genetic Testing in Prostate Cancer

Recognizing differences in prostate cancer germline mutations among ancestral groups will play important roles in improving diagnosis and treatment [20•]. However, there remains no consensus on which genes should be included in pan-ancestral germline genetic testing panels, or screening guidelines following genetic testing. The Natural History Study of Men at High Genetic Risk for Prostate Cancer (ClinicalTrials.gov NCT03805919) is an important ongoing trial to establish screening recommendations after patients undergo genetic testing. This trial is enrolling men from 30 to 75 years of age without prostate cancer and with one or more recorded likely pathogenic or pathogenic germline variants within coding regions of prostate cancer-related risk genes: *BRCA 1 and 2*, DNA Mismatch Repair (MMR) genes associated with Lynch syndrome (*EPCAM*, *MLH1*, *MSH2*, *MSH6*, *PMS2*), *ATM*, *BRIP1*, *CHEK2*, *FANC* (*FANCA*, *FANCB*, *FANCC*, *FANCD2*, *FANCE*, *FANCF*, *FANCG*, *FANCI*, *FANCL*, and *FANCM*), *HOXB13*, *NBN*, *PALB2*, *RAD51C*, *RAD51D*, or *TP53*. It remains to be seen if testing these mutations will help to improve recommendations for genetic testing among men of AA, EA, and other ancestry.

Emerging data highlights the prevalence of DDRG mutations among the different ancestral groups [21]. The genetic aberrations can also be associated with disease severity and could potentially help explain why African American men are more prone to aggressive prostate cancer. Kohaar et al. show that specific mutations in PMS2, BRCA1, and the genes of the RAD family (RAD51, RAD54L, RAD54B) are more frequent and associated with higher rate of metastasis in AA patients when compared to EA patients from an equal-access healthcare system [20•]. Additionally, rare variants of HOXB13, GPRC5C, and IGF1R genes were recently reported in men of West African men and AA [22–25]. Among these germline mutations, the specific HOXB13 mutation was associated with 2.4-fold increased odds of developing prostate cancer and greater risk of aggressive and advanced disease [22]. Overall, these findings provide impetus for more studies to discover and validate clinically significant and actionable germline mutations to enable germline genetic tests to perform equally well in diverse populations.

Currently, ancestry-informed testing for germline mutations is not a major consideration in the prostate cancer management guidelines, and so there is no clarification on which germline mutations to test based on the individual patient’s ancestry. Furthermore, the current testing panels focus on a limited number of germline pathogenic variants which are not frequently mutated in AA patients, thus leading to potentially false negative results especially with DDRG mutations in AA population. Importantly, Weise et al. pointed out that clinicians and genetic test providers should recognize the lack of equal testing rates among non-white men in germline testing cohorts [26]. This important factor might explain why non-white patients have higher rates of variants of uncertain significance (VUS) [26, 27]. Increasing the number of minorities in germline testing and related clinical trials is imperative for improved germline testing panels, screening/treatment stratification, and recommendations [28].

## Healthcare Inequalities in Prostate Cancer

It has become generally accepted that the underlying causes of health inequities are multi-factorial and include biological, structural, and social determinants [29]. In this context healthcare, genetic and biological differences appear to be impactful drivers of disparity in prostate cancer [30, 31]. Of note, the lack of inclusive genetic tests and genetic testing is an important challenge in health care. In particular, healthcare inequalities and their impact on the entire spectrum of care of prostate cancer need to be addressed in the context of men of AA [32]. Lowder et al. analyzed the potential interaction between socioeconomic status (SES), environmental status, and genetics on prostate cancer in men of AA [33••]. Their study compared access to healthcare and showed that while 9.6% of black men were uninsured in 2019, 16.7% of Hispanic men were uninsured, and 5.2% of non-Hispanic white men were uninsured, the rates of prostate cancer were the lowest in Hispanic men. They also report that while increase in SES correlated to a decrease in prostate cancer-specific mortality in white men, increase in SES did not have a decrease in mortality in black men. An analysis of 60,035 male veterans (30.3% black and 60.7% non-Hispanic White) in the equal-access VA healthcare systems showed a similar overall survival rate between the two ethnicities [34]. Dess et al. reported that population-level risk factors such as lower socioeconomic status, decreased insurance, and increased comorbidities in African Americans contribute to their increased mortality from prostate cancer [35]. In this study, outcomes for prostate cancer in the equal-access VA system were analyzed and showed that men of AA may have better outcomes than white men when receiving similar care and access to care. Sivakumar et al. showed that men of AA outside of an equal access to care system received a median of two lines of therapy prior to complete genomic profiling (CGP), compared to one line of therapy in EA men. After CGP, only 5% of AA men were offered a clinical study drug, while 85% of men of EA were offered a clinical study drug [30]. Lower SES and decreased access to transportation and healthcare for men of AA men likely contribute to the low referral and participation rates in clinical trials. One study showed that in the USA, only 2.9% of patients in metastatic castration-resistant prostate cancer clinical trials were men of AA, and other minorities composed less than < 0.5% [35].

There is a general agreement that disparities in healthcare exist within the general population of the USA and disparities exist in morbidity and mortality in prostate cancer. While equalizing access to care can influence survivability, the incidence of prostate cancer in men of AA is due to more than social factors. Increasing the proportion of ancestral groups in genetic databases, recruitment, and participation of men of diverse ancestral background in clinical trials and education to providers and genetic counselors on incorporation of ancestral considerations will improve overall outcomes for prostate cancer [16, 36–38].

## Conclusions

While germline genetic screening and counseling are guideline-supported for certain men with prostate cancer diagnoses, it is variably performed for many reasons, including lack of understanding of the impact of germline mutations in treatment consideration, lack of access to timely genetic counselors, perceived lack of time or expertise to address findings of genetic testing, and a gap in our knowledge of ancestry-associated mutations especially in the context of DDRGs. There is rapidly emerging evidence in ancestral differences in germline mutations and the role of these mutations in prostate cancer pathogenesis. Further study of ancestry-informed genetic risk and how to effectively implement genetic screening in a multidisciplinary setting has potential for a significant impact on clinical management through guiding recommendations for screening, genetic counseling, implications for family members, and treatment and clinical trial enrollment.

## Declarations

### Disclaimer

The contents of this publication are the sole responsibility of the author(s) and do not necessarily reflect the views, opinions, or policies of Uniformed Services University of the Health Sciences (USUHS), the Henry M. Jackson Foundation for the Advancement of Military Medicine, Inc., the Department of Defense (DoD), or the Departments of the Army, Navy, or Air Force. Mention of trade names, commercial products, or organizations does not imply endorsement by the US Government.
